# The Molecular Pathology and Clinical Impact of HBV Genetic Variability

**DOI:** 10.1155/2011/242106

**Published:** 2011-11-09

**Authors:** Isabelle Chemin, Heléne Norder, Patrick Soussan, Runu Chakravarty

**Affiliations:** ^1^INSERM U1052, Institut National de la Santé et de la Recherche Médicale and Université Lyon, Lyon 69003, France; ^2^Section for Hepatitis and Enteroviruses, Department of Virology, Swedish Institute for Communicable Disease, Solna, Sweden; ^3^INSERM U845, 156 Rue de Vaugirard, 75015 Paris, France; ^4^Indian Council of Medical Research Virus Unit, Kolkata, New Delhi 110029, India

According to the World Health Organization, about 2 billion persons worldwide have been infected with hepatitis B virus (HBV). More than 350 million are chronically infected worldwide with a high risk of developing liver cirrhosis and hepatocellular carcinoma (HCC) (representing 1 million deaths per year). The epidemiology of HCC is peculiar with both geographic and temporal patterns of incidence paralleling exposure to viral etiologic factors. The highest HCC incidence rates are areas endemic for chronic infection with hepatitis B virus (Asia and Africa). Based on sequence divergence in the entire genome of >8%, HBV genomes have been classified into eight groups designated from A to H. The genotypes of HBV have distinct geographical distributions. Although preliminary clinical studies seem to indicate that there is an association between HBV genotype and natural history of infection and/or response to antiviral therapy, further evaluations on larger collectives of patients are necessary to give a clearer picture of the subject. The first paper of this special issue addresses the aetiology and picture of CTNNB1 and TP53 mutations in Thailand patients with HCC and provides a detailed study of the situation in an Asian country. The second paper presents the study on the etiology and viral genotypes in Colombia; these data are not common with South America. The two subsequent papers address the question of HBV variability and HCC in two different countries and continents. Indeed, the third paper is on TP53 mutations and HBx gene of hepatitis B virus status in HCC in Iran. It is bringing interesting discussion about the potential link between HBV genotypes and TP53 mutation R249S. The fourth paper of this special issue presents a fully picture of viral hepatitis viruses and TP53 mutations in HCC from Columbia. The fifth paper describes the evolution of HBV “quasispecies” in a chronic HBV chronic carrier over a 2-year period. The final paper of this special issue proposes an original study on the importance of HBV variability on RNAi strategies.

*Isabelle Chemin* *Isabelle Chemin* *Heléne Norder* *Heléne Norder* *Patrick Soussan* *Patrick Soussan* *Runu Chakravarty* *Runu Chakravarty*

